# The influence of occupational stress factors on the nicotine dependence: a cross sectional study

**DOI:** 10.1186/1617-9625-8-6

**Published:** 2010-04-13

**Authors:** Anna Schmidt, Melanie Neumann, Markus Wirtz, Nicole Ernstmann, Andrea Staratschek-Jox, Erich Stoelben, Jürgen Wolf, Holger Pfaff

**Affiliations:** 1 Institute for Medical Sociology, Health Services Research and Rehabilitation Science (IMVR), Faculty of Human Science and Faculty of Medicine, University of Cologne, Eupener Strasse 129, Cologne 50933, Germany; 2 Integrated Curriculum for Anthroposophic Medicine, private University of Witten/Herdecke, Gerhard-Kienle-Weg 4, Herdecke 58313, Germany; 3 Institute for Psychology, University of Education Freiburg, Kunzenweg 21, Freiburg 79117, Germany; 4 LIMES (Life and Medical Sciences Bonn), Program Unit Molecular Immune & Cell Biology, Functional Genomics, University of Bonn, Karlrobert-Kreiten Strasse 13, Bonn 53115, Germany; 5 Hospital Merheim, chest clinic, Ostmerheimer Str. 200, Cologne 51109, Germany; 6 First Department of Internal Medicine, Molecular Tumour Biology and Tumour Immunology & Centre for Integrated Oncology (CIO), University Hospital Cologne, Kerpener Strasse 62, Cologne 50937, Germany

## Abstract

**Objective:**

This study analyses the association between occupational stress factors and nicotine dependence. Our hypothesis is that occupational stress factors increase nicotine dependence.

**Methods:**

Data were taken from the Cologne Smoking Study, a case-control study that examines which genetic/psychosocial factors lead to a higher risk for smokers to suffer from cardiac infarction, lung cancer and/or to become addicted to nicotine. Our sample consisted of N = 197 currently smoking and employed participants. Nicotine dependence was measured using the **F**agerström **T**est for **N**icotine **D**ependence (**FTND**). The extent of the stress factors experienced at work was assessed using the **E**ffort-**R**eward **I**mbalance scale (**ERI**). Logistic regression was used for the statistical analysis.

**Results:**

Contrary to our hypothesis, the results show that occupational stress factors are actually associated with lower levels of nicotine dependence (N = 197; adjusted OR = 0.439; p = .059).

**Conclusions:**

One possible explanation for the study's findings is that the participants have a heavy workload and can only smoke in their spare time. Another reason may be workplace smoking bans. Furthermore, the Fagerström Test for Nicotine Dependence is unable to examine nicotine dependence during working hours.

## Introduction

Tobacco use is a risk factor for six of the eight leading causes of death. In fact, tobacco kills a third to half of all its users. On average, every tobacco user loses 15 years of their life. The total number of tobacco-attributable deaths - from ischaemic heart disease, lung cancer and other diseases [1] - is projected to rise from 5.4 million in 2004 to 8.3 million in 2030 [2].

Nicotine dependence and the degree of that dependence are determined by individual, genetic and psychosocial factors as well as combinations of these factors [3]. Psychosocial factors, both occupational (e.g., work stress) and personal (e.g., poor quality of life), have an influence on the initiation and extent of smoking [4,5]. For example, smoking is used as a coping strategy for dealing with work stress [6,7]. The degree of cigarette consumption can therefore shed some light on potential stress at work. By using the "**F**agerström **T**est for **N**icotine **D**ependence" (**FTND**), it is possible to obtain more detailed information about survey participants' smoking behaviour than simply asking them to provide their smoking status. The FTND is an internationally recognized and statistically validated instrument for assessing the degree of nicotine dependence in smokers and has been tested in numerous empirical studies [8].

The effort-reward imbalance model provides a theoretical approach to assessing psychosocial stress experienced at work as measured by the **E**ffort-**R**eward **I**mbalance (**ERI**) scale [9,10]. This approach has been successfully tested and examined in many social epidemiological studies [11]. By measuring psychosocial stress at work, it is possible to identify a risk group and to then intervene using measures targeted at that particular group [12].

Originally developed to explain the adverse health effects of stressful work experiences, the ERI model posits that effort at work is exerted as part of a socially organized exchange process, to which society at large contributes through occupational rewards. These rewards are distributed by means of three transmitter systems: money, esteem and job security/career opportunities. The model claims that an imbalance between high efforts and low rewards may cause a state of emotional distress [13]. In addition to the two work-related dimensions of effort and reward, overcommitment at work acts as a personal risk factor. Separate and combined effects of these three dimensions on health are then postulated [13].

Only one other study has used the FTND to determine whether there is correlation between nicotine dependence and job stress factors measured using the Karasek model of job strain [14]. Other social epidemiological studies support the hypothesis of a correlation between job stress factors and nicotine dependence by using the ERI scale and data on smoking status [8,13,15,16].

Two cross-sectional studies conducted by Ota and colleagues (2004) [17] and John (2006) [14] are the only studies that support a different thesis. These studies, which used the FTND, found that smoking is unrelated to job stress.

Our study aims to determine whether there is a correlation between the experiences of occupational stress, measured using the ERI scale, and nicotine dependence, measured with the FTND. A systematic search in PubMed in January 2008 (MeSH terms: *disorder, imbalance, psychosocial factor(s), working stress, effort, reward, gratification crisis, worker, nicotine dependence and smoking*) found no other studies that have investigated this research question using both of these same measures.

## Methods

### Study design and participants

Data for the study were taken from the **Co**logne **Sm**oking **S**tudy (**CoSmoS**), a case-control study that examines which genetic/psychosocial factors lead to a higher risk for smokers to suffer a cardiac infarction, develop lung cancer and/or become addicted to nicotine. The study was approved by the Ethics Committee of the University Hospital of Cologne (UHC). Patients were included in the study after signing an informed consent form.

CoSmoS consisted of N = 524 participants. Of these participants, 457 (87.2%) were smokers/ex-smokers and 64 (12.8%) were non-smokers. The study's design required that primarily smokers be included in the study. 180 lung cancer patients and 170 myocardial infarction patients (acute myocardial infarction and/or a history of myocardial infarction) were recruited at the UHC and the Chest Clinic Merheim. 174 control patients, who had not been diagnosed with either condition and who had not been admitted with a diagnosis of cancer and/or a nicotine-related disease, were selected from the Orthopaedics and Dermatology departments. The participants were surveyed in hospital in face-to-face interviews.

### Measures

Nicotine dependence was assessed using the FTND, a psychometrically evaluated instrument used to determine the degree of cigarette consumption and the inability to abstain from nicotine use [8,18,19].

The independent variable was measured using the German version of the ERI scale [13,9], which consists of three subscales: "effort," "reward" and "overcommitment". To evaluate the effort-reward imbalance experienced by study participants, only the scores of the effort and reward scales were needed; an effort-reward ratio was then computed using a standardized syntax [13].

The six items of the "effort" scale measure extrinsic components of stressful experiences at work. The response options for the "effort" scale are: "Disagree," "Agree, but I am not at all distressed," "Agree, I am somewhat distressed," Agree, I am distressed," and "Agree, I am very distressed". The "reward" scale includes 11 items assessing the extrinsic components of occupational rewards and contains questions pertaining to opportunities for advancement, employee appreciation, salary and job security. Participants with no superiors or colleagues have the option to respond with "Not applicable". For seven of the items, participants can respond with "Agree," "Disagree, but I am not at all distressed," "Disagree, I am somewhat distressed," "Disagree, I am distressed," and "Disagree, I am very distressed". The response options for the other four items are the same as those of the "effort" scale. The reliability and validity of the "effort," "reward" and "overcommitment" subscales as well as of the "effort-reward ratio" have been demonstrated in many studies [for an overview, see [12]].

### Statistical analysis

The individual items of the FTND were combined into a sum score for the multivariate analysis. Scores of one to three represent smokers with low nicotine dependence and scores of four to five represent smokers with a heavy dependence on nicotine [18,19]. In order to compare workers with low nicotine dependence to those with heavy dependence, the FTND sum score was dichotomised at the value of 4 (highly dependent) for the logistic regression because the dependent variable was not normally distributed.

The ERI analysis was conducted as follows: If the participants disagreed with a statement, their response was assigned a value of 0. If the participants agreed with a statement but did not experience any stress, their response was assigned a value of 1. The greater the level of stress experienced, the greater the value up to 4. The ERI is, therefore, a five-point Likert scale. When interpreting the values, the higher the sum score of the "effort" and "reward" subscales, the greater the level of occupational stress. Values over 1 indicate an imbalance between effort and reward [20].

A logistic regression model was calculated using all sociodemographic variables. Statistical data were analysed using SPSS version 15.0 for Windows.

## Results

### Descriptive statistics

The study sample consisted of N = 197 currently smoking and employed participants, of which 70 were lung cancer patients, 53 were myocardial infarction patients and 74 were control patients. To prevent any memory-based distortions, 64 non-smokers and 263 unemployed and retired patients were excluded from the study. The resulting subsample is representative of the original total sample of 524 patients because the study participants are evenly distributed between the two case-study groups and the control group and because all of the patients were hospitalized at the time of the survey. The distribution of the sociodemographic characteristics is shown in Table 1.

**Table 1:** Sociodemographic characteristics of the sample (N = 197).

Variables	N	%
**Sex**		
male	133	67.5
female	64	32.5

**Age**		
> 53	99	50.3
< 53	98	49.7

**Family status**		
not married	53	26.9
married	144	73.1

**Religion**		
not religious	63	32.0
religious	134	68.0

**Level of education**		
low	125	63.5
high	72	36.5

**Residence**		
country	121	61.4
city	74	37.6

The following degrees of nicotine dependence were found among the study sample: 51 participants (25.9%) had a very low dependence on nicotine, 54 (27.4%) had a low dependence, 26 (13.2%) were moderately dependent, 45 (22.8%) were highly dependent and 21 (10.7%) were very highly dependent [21]. The mean value of the FTND scale was 2.65 (range: 1-5), which indicates a moderate level of dependence. After dichotomization, there were 131 workers with low nicotine dependence and 66 with high dependence.

Results of the ERI scale showed that 13.8% of participants do not experience an imbalance between effort and reward (up to a value of 0.99). 67.2% experience a low imbalance (values of 1 to 1.99), 16.9% experience a moderate imbalance (values of 2 to 2.99) and only 2.1% experience a high imbalance (values of 3 to 4). The mean value of the effort-reward ratio is 2.07 (range: 2-3).

### Multivariate analysis

The results of the logistic regression are shown in Table 2.

**Table 2:** Results of the logistic regression model, nicotine dependence and ERI (N = 197).

Independent variable	Beta	SC	SD	p-value	OR	95% CI	
						
						lower limit	higher limit
Sex (female/male*)	-0.460	1.567	.368	.211	0.631	0.307	1.298
Age (> 53/< 53*)	-0.443	1.735	.337	.188	0.642	0.332	1.241
Family status (married/not married*)	-0.727	3.980	.364	**.046**	0.483	0.237	0.987
Religion (religious/not religious*)	-0.755	4.681	.349	**.030**	0.470	0.237	0.931
Level of education (high/low*)	-0.896	5.544	.381	**.019**	0.408	0.193	0.861
Residence (city/country*)	0.475	1.947	.341	.163	1.609	0.825	3.137

ERI (no effort-reward imbalance/effort-reward imbalance*)	0.822	3.572	.435	.059	0.439	0.187	1.031

Cox and Snell pseudo-R^2 ^= .109							
Nagelkerke pseudo-R^2 ^= .151							
Mc Fadden pseudo-R^2 ^= .10							

*Note*: * = reference group, Beta = regression coefficient, SC = standardized effect coefficient, SD = standard deviation, CI = 95% confidence interval

For currently smoking and employed participants, a decrease (p = .059) in their likelihood to suffer from nicotine dependence was found to be associated with their experience of an effort-reward imbalance (adjusted OR = 0.439; CI = 0.187-1.031). The amount of explained variance in this model is 15.1% (Nagelkerke pseudo-R^2^; for the other coefficients, see Table 2, rows 6 and 8). The specificity of the model is 65.6% and the sensitivity is 57.6%.

The model also demonstrates that being religious, being married and having a higher level of education have a significant effect on the prevention of nicotine dependence (Table 2).

## Conclusions

### Main findings

Contrary to our hypothesis, the analysis indicates that the experience of occupational stress factors reduced the likelihood of nicotine dependence in currently smoking and employed participants. The study conducted by Ota and colleagues (2004) [16] and John (2006) [15], as mentioned above, supports our finding that smoking is unrelated to job stress. In scientific literature, both hypotheses have been discussed and debated. For example, contrary to the findings of our study, Kouvonen and colleagues found that Finnish public sector employees who experience an effort-reward imbalance at work are subject to an increased risk of regular tobacco consumption [7].

Given the marginal significance of the association found between nicotine dependence and occupational stress in our study, caution should be taken when drawing conclusions from its findings. Our analysis shows that heavy employee workload is associated with lower nicotine dependence. One possible explanation for this is that a heavy workload may drive employees to smoke in their spare time only. Another reason may be the growing number of workplace smoking bans leading participants to reduce their consumption [22]. A further possibility is that the Fagerström Test for Nicotine Dependence is not fully able to examine nicotine dependence during working hours.

The logistic regressions in our study also indicate that not being religious, not being married and having a lower level of education are significant risk factors for nicotine dependence. These findings correspond to those of Blay and colleagues (2008) [23], who found that evangelical affiliation reduced the odds of being a tobacco user by 51%. It is therefore possible to assert that religious affiliation is associated with a decrease in the frequency of tobacco usage [24].

The finding that a higher level of education is a protective factor against nicotine dependence may be explained by the fact that those with a higher level of education are aware of the risks of smoking and belong to the group of people among whom smoking is less common [25].

A possible explanation for the finding that being married is a protective factor against nicotine dependence is the fact that people who are in a relationship tend to take care of each other [26].

### Limitations of the study

Due to the retrospective design of our study, there may be memory-based distortions in the participants' responses. In addition, the first author, who was responsible for interviewing participants in CoSmoS, noticed that the questions of the ERI scale evoked emotional reactions of denial and reticence in the participants, making it difficult for them to respond.

Further, because data were collected in face-to-face interviews, the presence of another individual at these interviews (e.g., patient, visitor) may have been enough to distort the results [27]. Social desirability also seemed to play a major role in the response behaviour of the participants. Because "social desirability bias" involves the systematic distortion of responses in a certain direction, contorted marginal distributions in the participants' responses must be considered when looking at the results [28].

Unlike the studies discussed above, the CoSmoS study surveyed severely ill participants. Interviews therefore had to be conducted within the hospital and were not anonymous. Also, since this was a correlative cross-sectional study, only associations could be examined. Furthermore, this retrospective survey was probably an underpowered substudy of a heterogeneous population.

### Future research

Both the findings of previous studies as well as the findings of the present study indicate the need for further investigations. Future research should include prospective studies with larger samples of currently smoking and employed individuals from various professional fields. The FTND is not fully able to examine employee dependence during working hours. Future studies should aim to obtain a more precise assessment of employee smoking behaviour at work. The growing number of workplace smoking bans may be pushing employees to shift their smoking habits into their spare time. Items which take this shift into consideration may be a reasonable supplement to the evaluation instrument.

The sizes of the individual case-study groups in this study were too small for studying and comparing the experience of work stress among the lung cancer patients, patients with myocardial infarction and the control group. Due to the small sample size, the number of independent variables studied for their association with nicotine dependence had to be limited. An excess of parameters in comparison to the information content of the data, would have led to unstable regression coefficient estimates (i.e., "overfitting") [29]. In future studies, it would certainly be interesting to determine whether there is an association between work stress and nicotine dependence. However, a larger sample size would be needed.

### Policy implications

The results of this study indicate that employees who experience stress at work are more likely to have a low dependence on nicotine. It, therefore, seems impossible to provide any policy implications because it cannot be said that employees who do not experience work stress have higher nicotine dependence or that greater stress at work results in lower nicotine dependence.

Although our study as well as that of Blay and colleagues (2008) [23] show that being religious, being married and having a higher level of education are protective factors against nicotine dependence, it is impossible to derive policy implications because these three factors cannot be influenced directly.

## Competing interests

The authors declare that they have no competing interests.

## Authors' contributions

AS was the lead author of the manuscript. HP, MN, AS-J, ES and JW participated in the design of the study. Working together with three other research assistants, AS collected the data in face-to-face interviews. MW directed the statistical analyses. NE prepared the analyses and the tables. All authors read and approved the final manuscript.
